# Cholesteatoma gene expression of matrix metalloproteinases and their inhibitors by RT-PCR

**DOI:** 10.1590/S1808-86942012000300019

**Published:** 2015-10-14

**Authors:** Carlos Eduardo Borges Rezende, Ricardo Peres do Souto, Priscila Bogar Rapoport, Laís de Campos, Marcela Bovo Generato

**Affiliations:** a MSc student in the Health Sciences Program at FMABC (Professor of otorhinolaryngology at FMABC).; b PhD in Biochemistry at USP (Associate Professor of Biochemistry at FMABC).; c PhD in Medicine at the University of São Paulo (Professor of Otorhinolaryngology at FMABC).; d Student of Pharmaceutic Sciences at FMABC (Student of Pharmaceutic Sciences at FMABC).; e Student of Pharmaceutic Sciences at FMABC (Student of Pharmaceutic Sciences at FMABC).

**Keywords:** cholesteatoma, gene expression, matrix metalloproteinases, middle ear, tissue inhibitor of metalloproteinases

## Abstract

Acquired middle ear cholesteatoma is a benign keratinizing hyperproliferative squamous epithelial lesion that may result in the destruction of the bone structures surrounding the temporal bone. Recent studies show that variations in cellular production of matrix metalloproteinases (MMPs) and their specific inhibitors (TIMPs) contribute to the pathophysiology of cholesteatoma.

**Objective:**

This study aims to analyze the use of RNA amplification tests to evaluate the expression of MMP and TIMP isoforms in cholesteatomas and their correlation with disease severity.

**Materials and Methods:**

This is a prospective study. Nineteen cholesteatoma cases at different stages were selected. RNA collected from biopsy specimens was submitted to reverse transcription polymerase chain reaction (RT-PCR) for semiquantitative amplification of MMP2, MMP3, MMP9, MMP13 and TIMP1.

**Results:**

Six cholesteatomas were positive for at least one of the studied genes. Four samples amplified a single gene (MMP2 or MMP13) and two samples amplified three genes (MMP2, TIMP1 and MMP3 or MMP13). No sample amplified MMP9.

**Conclusion:**

RT-PCR can be used to assess MMP and TIMP gene expression in cholesteatomas despite technical difficulties. Gene expression profiles could not be related to disease severity.

## INTRODUCTION

Middle-ear acquired cholesteatoma is a benign squamous epithelial keratinizing hyperproliferative lesion resembling the epidermis^1^ that develops inside the tympanic cavity^2^, ^3^, ^4^. It manifests as a type of epidermoid cyst with an extracellular matrix made up of squamous stratified keratinized epithelium over a secondary perimatrix containing collagen and elastic fibers, fibroblasts, and inflammatory cells^5^, ^6^. Middle-ear cholesteatomas are characterized by intense cell proliferation - accompanied by the consequent accumulation of keratin debris - and destruction of bone structures surrounding the temporal bone. They may involve the ossicles, blood vessels, the facial nerve, and even invade the inner ear and intracranial space^7^, ^8^, ^9^. Cholesteatomas may lead to the onset of hearing loss, tinnitus, vertigo, loss of balance, and other severe complications such as meningitis, sigmoid sinus thrombosis, facial paralysis, and brain abscess^2^.

It has recently become evident that the proteolytic activity triggered by cholesteatomas plays a key role in the bone remodeling of the middle ear and the temporal bone^10^. Bone lysis and recurrence are relevant features in the pathophysiology of cholesteatoma, giving it the status of a dangerous, difficult-to-treat condition^11^. Alterations in keratinocyte proliferation, differentiation, and migration are impacted by fibroblast activation in the perimatrix and by the release of cytokines and growth factors by inflammatory infiltrate cells^4^, ^12^.

Injury to tissues adjacent to cholesteatomas also occurs due to the action of various proteolytic enzymes such as plasminogen activators and matrix metalloproteinases (MMPs)^3^, ^7^. MMPs are zinc and calcium-dependent endopeptidases synthesized by different types of cells such as fibroblasts, keratinocytes, macrophages, and endothelial cells activated by proteolytic cleavage^8^, ^10^, ^13^, ^14^, ^15^, ^16^. MMP proteolytic activity is precisely controlled by their precursors during activation and inhibited by endogenous inhibitors, alpha macroglobulins, and tissue inhibitors of metalloproteinases (TIMPs)^8^, ^17^. The balance between MMPs and TIMPs is critical in determining the integrity of the extracellular matrix (ECM); thus, the variations in presence and activity level of these proteins may contribute in a number of tissue events observed in cholesteatoma patients^3^, ^8^, ^17^.

Specific cholesteatoma MMP isoenzymes (MMP2, MMP3, and MMP9) were first identified in 1996^17^ with the use of immunohistochemistry tests. Since then, immunohistochemistry has been extensively used to analyze and compare changes in enzyme levels in cholesteatomas and healthy tissues. Increased levels of MMP9^7^, ^10^, ^11^, ^14^, MMP2^5^, MMP1^18^, MMP8 and MMP13^19^ have been reported. Immune labeling of MMP2, 3 and 9 was observed mainly in the basal and suprabasal layers of the cholesteatoma epithelium^10^, ^14^, ^17^; MMP9 was specifically seen in areas with inflammatory cell infiltration^8^.

This study aims to analyze the applicability of reverse transcription polymerase chain reaction (RT-PCR) to semiquantitatively assess gene expression in matrix metalloproteinases and their inhibitors. If RT-PCR is proven adequate, we will attempt to correlate gene expression to disease severity based on the patients' clinical history, audiometric assessment, and imaging findings.

## MATERIALS AND METHODS

This is a prospective study designed to offer the basis required to reach the proposed goals and additional information on the topic, aside from aiding in the identification of the determining factors connected to the phenomena and events described herein^20^. Nineteen patients aged between 5 and 70 years diagnosed with various stages of chronic cholesteatomatous otitis media were enrolled in this study. They were seen and operated at the institution's ENT ward from 2007 to 2009. Patients previously submitted to mastoidectomy and with unconfirmed diagnosis of cholesteatoma by pathology tests were excluded. This research project was approved by the Institution's Ethics Committee and granted permit nº. 356/2007.

Auditory involvement was assessed through pure-tone and speech audiometry and ranged from mild conductive dysacusis to profound sensorineural dysacusis. The extension of the involvement by cholesteatoma was analyzed in CT scans and graded based on findings such as scutum erosion, erosion of the ossicular chain, fistula in the lateral semicircular canal, erosion of the tegmen tympani, and Fallopian canal dehiscence. The extent of erosion was assessed by the number of involved structures. Presence of erosion on one structure was categorized as +; erosion on two structures was graded ++; and erosion on three or more structures was rated as +++. None of the patients had advanced stage complications such as facial paralysis, meningitis, encephalitis, or brain abscess.

Open mastoidectomy was the approach used to remove the cholesteatomas in our group of patients. Surgery findings on the extent of involvement and erosion supported the imaging findings on the integrity of the middle ear ceiling (tegmen tympani), the wall of the lateral semicircular canal, the scutum, and the facial canal (Fallopian canal).

**Chart 1:**
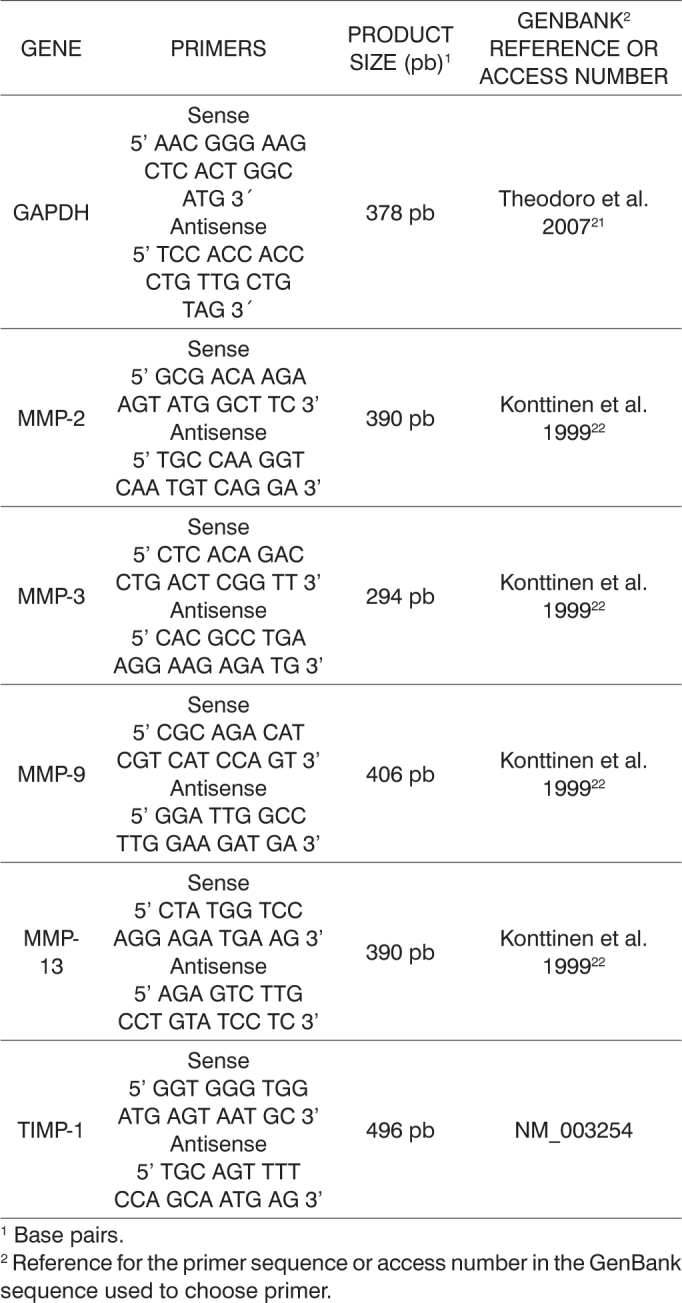
Characterization of the chosen primer pairs.

Nineteen suspicious specimens were collected and all were confirmed as cholesteatoma by the pathologist. The specimens were promptly frozen in liquid nitrogen and kept in a freezer at -70°C to maintain messenger ribonucleic acid (mRNA) integrity. RNA was isolated using Trizol^®^ (Invitrogen) or the Illustra RNAspin^®^ (GE Helthcare) kit. RNA quantification was done through UV spectrophotometry at 260 nm using device GeneQuant^®^ (Amersham-Pharmacia) or by fluorometry in a Qubit^®^ device (Invitrogen). Total RNA was transformed into complementary DNA (cDNA) by reverse transcription using oligonucleotide dT as a primer and enzyme Superscript RT III^®^ (Invitrogen). The specific cDNA of each MMP or TIMP isoform was amplified by PCR, using reagent mix Master Mix (Promega^®^) and the primers described in the literature (glyceraldehyde 3-phosphate dehydrogenase - GAPDH^21^ and MMP2, 3, 9 e 13^22^) or others picked based on their respective mRNA sequences found on GenBank (TIMP-1). For more data on primers, please refer to Chart 1. The amplification protocol is initialized with sample incubation at 94°C for 5 minutes, followed by 35 cycles at three temperatures: 94°C for 1 minute (denaturation), 58°C for 1 minute (annealing), 72°C for 1 minute (extension); the last step is incubation at 72°C for 10 minutes. PCR products were then separated by 2% agarose gel electrophoresis (buffer TAE, 100 V) with ethidium bromide added as a fluorescent tag. The gel images were captured using a digital camera.

## RESULTS

The clinical characterization of the cholesteatomas of the 19 patients enrolled in the study can be seen in Table 1. Patient mean age was 38.7 years (7-67). Most of them had right ear cholesteatomas (14/19; 73.7%). CT images indicated a wide array of injuries; one patient had no erosion, while five had erosion in only one ear structure; eight had two compromised structures and five had three structures affected by erosion. As for dysacusis, most patients had mixed hearing loss.

**Table 1:** Cholesteatoma clinical features and MMP and TIMP gene expression.

Patient	Age (years)	Dysacusis	Erosion	MMP-2	MMP-3	MMP-13	TIMP-1
1	12	Mild conductive	-	-	nt	nt	nt
2	27	Severe mixed	+++	+++	-	+	++
3	46	Mild conductive	+	+++	+	-	+
4	30	Severe mixed	+	-	-	-	-
5	7	Moderate conductive	+++	-	nt	nt	nt
6	52	Severe mixed	++	-	nt	nt	nt
7	54	Profound mixed	++	-	nt	nt	nt
8	58	Moderate mixed	+++	-	nt	nt	nt
9	17	Moderate sensorineural	++	-	nt	nt	nt
10	42	Moderate conductive	+	+	-	-	-
11	45	Severe mixed	++	-	-	-	-
12	37	Moderate conductive	++	-	-	-	-
13	67	Severe mixed	+++	-	-	-	-
14	42	Moderate conductive	+	-	-	-	-
15	14	Moderate conductive	+	-	-	++	-
16	42	Moderate conductive	+++	-	-	-	-
17	27	Mild conductive	++	-	nt	++	nt
18	63	Severe mixed	++	-	nt	nt	nt
19	43	Moderate conductive	++	-	nt	+	nt

nt: not tested.

**Figure 1:**
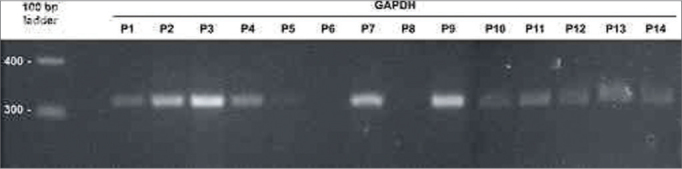
GAPDH amplification using the volumes fixed for RNA preparations. Analysis by 2% agarose gel electrophoresis dyed with ethidium bromide. Molecular weight standard = 100 bp ladder. P1-P14: patients 1 to 14.

The first procedure carried out to analyze gene expression was the amplification of the mRNA from gene GAPDH, assuming that there would be identical expression of this enzyme in the glycolytic pathway of all analyzed tissue samples. Despite the differences in RNA concentration in the samples, higher levels of homogeneity in GAPDH amplification yield were attained when sample equivalent volume were analyzed (Figure 1). GAPDH amplification from the sample collected from patient 9 was processed in 30, 35, and 40 PCR cycles. At 35 cycles, the set condition for amplification used in the study, the reaction had not reached saturation.

**Figure 2:**
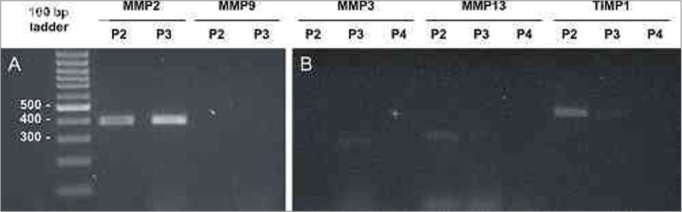
MMP and TIMP isoform amplification for selected samples. Analysis by 2% agarose gel electrophoresis dyed with ethidium bromide. Molecular weight standard = 100 bp ladder. (A) MMP-2 and MMP-9 for samples P2 and P3. (B) MMP-3, MMP-13, and TIMP-1 for samples P2, P3, and P4.

RT-PCR was applied to all samples for metalloproteinases 2 and 9; only samples 2, 3, and 10 had amplification for MMP2, and none had amplification for MMP9 (see results on Figure 2A). Samples 2,3,4,10,11,12,13,14,15, and 16 were amplified for MMP3, MMP13 and TIMP1; clear amplification for TIMP1 and MMP13 was seen in samples 2, 15, 17, and 19, and for MMP3 and TIMP1 in sample 3 (see Figure 2B). Some reactions were not successful due to limited availability of RNA and technical difficulties in processing small highly keratinized samples. The results for MMP and TIMP amplification can be seen on Table 1.

## DISCUSSION

Chronic cholesteatomatous otitis media is still a relevant public health concern in developing countries. It is an aggressive benign inflammatory disease that may evolve to hearing impairment as it compromises soft tissue, bone liners, ossicles and invades adjacent structures such as nerves, blood vessels, the cochlea, the labyrinth, and the brain.

The presence of MMPs and their inhibitors may be associated with higher levels of cholesteatoma-related lysis. RT-PCR may aid in the analysis of the expression of MMPs and their inhibitors and in the establishment of correlation between molecular activity and disease aggressiveness. Once such correlation is confirmed, it will be possible to propose strategies to control the disease's biochemical development. Some studies have found positive correlations between cholesteatoma aggressiveness and increased levels of MMP2^5^ and MMP9^10^, ^23^, given the way these enzymes operate biologically. MMP inhibition is being tested as a therapy for various ENT disorders^24^; however, in a pioneering study that looked into treating cholesteatomas in animal models with MMP-inhibitor ilomastat, disease progression remained unaffected^25^.

Our study found high levels of MMP2 expression in samples 2 and 3, and lower intensity expression in sample 10. Contrary to what various other authors have found^7^, ^10^, ^13^, ^23^, enzyme MMP9 was negative for all samples in our trial. It should be noted that the MMP9 primer pair amplifies human RNA^22^. Additionally to MMP2, gene expression was found for MMP3 (sample 3), MMP13 (samples 2, 15, 17, and 19), and TIMP1 (samples 2 and 3). Presence of MMP3, MMP13, and TIMP1 in cholesteatoma cells had been reported previously^3^, ^16^, ^13^.

Despite the progress observed in nucleic acid analysis techniques, only two papers in the specific literature have used RT-PCR to look into gene expression of MMPs and their inhibitors. The lack of papers on the presence of MMP and TIMP mRNA in cholesteatomas may be explained by the technical difficulties inherent to processing RNA from cholesteatoma specimens also experienced in this study.

The detection of MMP isoenzymes by histochemistry or RT-PCR does not imply that the enzymes are active, as metalloproteinases are synthesized in an inactive form. They become active catalysts only after partial proteolysis, i.e., they are zymogens^10^. MMP enzymatic activity in biologic samples is verified in a laboratory setting through separation by electrophoresis followed by gel hydrolysis in a technique called zymography. This is a complex standardization technique, fraught with conflicting results from different research groups. Such issue was observed for MMP2, as the zymograms in two papers failed to verify increased enzymatic activity in cholesteatoma fragments^10^, ^26^, while more recent studies carried out by three other groups detected gelatinolytic activity in the isoenzyme^1^, ^8^, ^16^. Controversy also looms over MMP9 analysis: increased activity was reported in two studies^8^, ^10^ and baseline activity in one other paper^13^. Additionally to these two isoforms, MMP1 and MMP3 activity in cholesteatomas were also assessed through zymography, and increased activity was seen only in the first isoenzyme^13^.

A literature review on MMP alteration in cholesteatomas reveals that there still is much to be explored in characterizing these enzymes as disease markers. The use of RT-PCR with such end has been almost completely disregarded until recently. This technique could be further utilized to quickly analyze the various yet unexplored isoforms from the MMP family that could have altered expression in cholesteatomas. And TIMP inhibitors have been barely studied to date, despite their role in controlling MMP activity.

Gene expression analysis by RT-PCR of matrix metalloproteinases and their inhibitors offers significant technical difficulties. Studies comprising larger numbers of patients or considering a greater number of variables might allow for stronger clinical correlations with the expression of these genes in cholesteatomas.

## CONCLUSION

Gene expression of matrix metalloproteinases and their inhibitors has been verified in cholesteatomas. RT-PCR detected mRNA in MMP2, MMP3, MMP3, and TIMP1, but not in MMP9. However, differences in MMP and TIMP expression associated with disease aggressiveness were not found.
